# Breastfeeding Duration and Cognitive Performance Among Youths

**DOI:** 10.1001/jamanetworkopen.2026.8725

**Published:** 2026-04-23

**Authors:** Xiaoqian Tang, Yuqing Qiu, Zhouyi Qin, Xin Yang, Junkai Luo, Jiaqi Hong, Yingying Guo, Nan Zhang, Shiying Lyu, Rujing Hu, Xiaoming Peng, Wei He

**Affiliations:** 1 Department of Nutrition and Food Hygiene, Children’s Hospital, Zhejiang University School of Medicine, National Clinical Research Center for Children and Adolescents’ Health and Diseases, Hangzhou, Zhejiang, China; 2 Chronic Disease Research Institute, School of Public Health, School of Medicine, Zhejiang University, Hangzhou, Zhejiang, China

## Abstract

**Importance:**

Evidence on the association between breastfeeding duration and cognitive development remains inconsistent, partly due to confounding by socioeconomic status (SES). Whether this association exists across populations with distinct socioeconomic patterning of breastfeeding is unclear.

**Objectives:**

To investigate the association between breastfeeding duration and cognitive outcomes among youths in China.

**Design, Setting, and Participants:**

This cross-sectional analysis used data from the China Family Panel Studies for youths aged 10 to 15 years who underwent cognitive assessments in 2010, 2014, or 2018 and were born between 1995 and 2009. Data were analyzed from February 2025 to January 2026.

**Exposures:**

Breastfeeding duration (≤6 months vs >6 months and continuous).

**Main Outcomes and Measures:**

Cognitive performance was assessed using mathematics and word recognition tests and was standardized as age-adjusted *z* scores. Poor cognitive performance was defined as scoring at or below the 15th percentile. Survey-weighted generalized linear models were fitted with progressive adjustment for demographic, perinatal, and socioeconomic factors. Restricted cubic splines were used to explore potential nonlinear associations.

**Results:**

Among 5436 youths included in the study (mean [SD] age, 12.03 [1.58] years; 52.5% male), 750 (13.8%) were breastfed for 6 months or less and 4686 (86.2%) for longer than 6 months. Longer breastfeeding duration was more common among youths from lower-income households (1229 [26.2%] vs 133 [17.7%]) and those with parents of lower educational attainment (maternal, 1464 [31.2%] vs 181 [24.1%]; paternal, 810 [17.3%] vs 99 [13.2%]). In unadjusted analyses, breastfeeding duration was not associated with mathematics (β = 0.03; 95% CI, −0.06 to 0.13) or word recognition (β = −0.02; 95% CI, −0.13 to 0.09) z scores. In models adjusted for SES, breastfeeding for more than 6 months was associated with higher mathematics (β = 0.14; 95% CI, 0.05-0.22) and word-recognition (β = 0.12; 95% CI, 0.02-0.21) *z* scores and lower odds of poor mathematics (odds ratio [OR], 0.65; 95% CI, 0.48-0.88) and word recognition (OR, 0.64; 95% CI, 0.47-0.87) performance. Restricted cubic spline analyses indicated that the associations may not be strictly linear.

**Conclusions and Relevance:**

In this cross-sectional study of Chinese youths, longer breastfeeding duration was not associated with cognitive performance in the unadjusted model but was associated with better cognitive performance after adjustment for SES. Longer breastfeeding was more common among families with low SES. Continued policy support, such as extended maternity leave and workplace lactation accommodations, may be warranted alongside further longitudinal research.

## Key Points

**Question:**

Is longer breastfeeding duration associated with better cognitive performance among youths aged 10 to 15 years in China?

**Findings:**

In this cross-sectional study of 5436 Chinese youths, breastfeeding for more than 6 months vs 6 months or less was not associated with youth cognitive performance in the unadjusted model but was associated with better cognitive performance after adjustment for socioeconomic status (SES). Longer breastfeeding was more common in households with lower SES.

**Meaning:**

These findings suggest no association between breastfeeding duration and youth cognitive performance in China unless confounding by SES is considered.

## Teaser

This cross-sectional study examines the association between breastfeeding duration and later cognitive performance among youths aged 10 to 15 years in China.

## Introduction

Breastfeeding is widely recognized as the optimal form of infant nutrition, and the World Health Organization (WHO) recommends exclusive breastfeeding for 6 months, followed by continued breastfeeding for at least 2 years.^1,2,3,4,5,6^ Despite global advocacy efforts, sustained breastfeeding rates remain below target levels.^7,8,9^ In addition to nutritional benefits, breastfeeding has been associated with child health and neurodevelopment.^10,11,12^

However, evidence regarding the association between breastfeeding duration and later cognitive performance remains inconsistent.^13^ A major methodologic challenge in interpreting this association is the confounding by socioeconomic status (SES).^14,15,16,17,18,19,20,21,22^ In many high-income countries, families with higher SES are more likely to breastfeed for longer durations, making it difficult to disentangle the independent association of breastfeeding from correlated advantages in the home and educational environment.^14,15^ By contrast, evidence from some low- and middle-income countries suggests varied associations between SES and breastfeeding duration, yet results regarding cognitive outcomes remain heterogeneous.^14,15,21,23^ Furthermore, studies on cognitive performance in adolescence remain limited.^24^

In China, rapid socioeconomic changes have resulted in distinct breastfeeding distributions across the population. Using nationally representative data from the China Family Panel Studies (CFPS), this study aimed to examine whether breastfeeding duration was associated with cognitive performance in adolescence after accounting for socioeconomic factors, leveraging the inverse socioeconomic-breastfeeding pattern observed in China during the study period. We further specified the a priori hypothesis that longer breastfeeding duration would be associated with better cognitive test performance and that this association would persist after adjustment for socioeconomic indicators.

## Methods

### Data Source

This cross-sectional study used data from the CFPS, a nationwide biennial longitudinal survey conducted by the Institute of Social Science Survey at Peking University. Launched in 2010, the CFPS covers 25 of 31 provinces (or municipalities) in China and follows approximately 16 000 households and their members based on reported family relationships, without verification of biologic or adoption status. The survey includes detailed information on cognitive functioning, socioeconomic characteristics, and education. Additional details on the CFPS are publicly available.^25^ Ethical approval was obtained from the Peking University Biomedical Ethics Review Committee, and written informed consent was obtained for broad research purposes. This secondary analysis of deidentified data followed the Strengthening the Reporting of Observational Studies in Epidemiology (STROBE) reporting guideline for cross-sectional studies.

From February 2025 to January 2026, we analyzed data from 3 CFPS waves conducted in 2010, 2014, and 2018. Breastfeeding duration was collected retrospectively through the child questionnaire, whereas cognitive assessments were administered to participants aged 10 years or older. Each participant contributed cognitive test scores from a single survey wave, defined as the first wave in which they were aged 10 to 15 years and completed both cognitive tests. We excluded those with missing breastfeeding information or who were never breastfed to align with the prespecified exposure definition. We also excluded individuals with missing covariates or survey design variables.

### Exposure Definition

Breastfeeding duration was assessed in the questionnaire using the first valid caregiver-reported response to reduce potential recall bias based on the item assessing total months of breastfeeding since birth (“Up to the present, how many months has the child received breast milk?” in 2010 or cumulative months after confirming cessation in later waves). This measure captured any breastfeeding, including exclusive and mixed feeding, regardless of the milk source (biologic mother or donor). For the primary analyses, breastfeeding duration was categorized as 6 months or less or longer than 6 months, consistent with the WHO recommendations and prior literature.^26^ For secondary analyses of temporal trends and potential nonlinear associations, breastfeeding duration was modeled as a continuous variable. The distribution of retrospectively reported breastfeeding duration is shown in eFigure 2 in Supplement 1.

### Outcome Definition

Cognitive performance was assessed using standardized mathematics and word recognition tests administered in the CFPS that were specified a priori as the core cognitive outcomes available for individuals aged 10 years or older. These tests measured crystallized intelligence and were adapted from Chinese primary and secondary school curricula. An adaptive testing format was used, with testing discontinued after 3 consecutive incorrect responses. Scores reflected the most difficult item answered correctly. Word recognition scores ranged from 0 to 34 and mathematics scores from 0 to 24, with higher scores indicating better performance. Given that cognitive performance varies with age during early adolescence, raw scores were converted to age-standardized *z* scores to improve comparability. These instruments have demonstrated adequate reliability and validity in prior studies of Chinese children.^27,28^ Outcomes were analyzed as both continuous *z* scores and as binary indicators of poor performance, defined as scores at or below the 15th percentile for each test.^29,30^

### Covariate Definition

Covariates were specified a priori based on prior literature and substantive considerations relevant to breastfeeding and child cognitive development.^14,17,31^ A directed acyclic graph was constructed to clarify assumed structural relationships and identify a minimal sufficient adjustment set for covariate selection (eFigure 3 in Supplement 1).^32^

Child-level variables included sex, ethnicity (Han or ethnic minority group), gestational age (≤9 or >9 months), birth order, and birth year (1995-1998, 1999-2002, 2003-2006, or 2007-2009). Ethnicity was reported by parents or guardians and categorized as Han vs. ethnic minority (including Buyi, Korean, Manchu, Miao, Yi, and other groups with few numbers reported) due to small individual group sizes. Ethnicity was included as a sociodemographic covariate reflecting social and cultural differences. Parent-level covariates included maternal and paternal age at birth (<30 or ≥30 years) and educational attainment (<6, 6-9, or >9 years). Household-level covariates included residence (urban or rural), primary cooking fuel type (clean or unclean), and annual household income per capita, categorized into low, middle, and high groups using the 25th and 75th percentiles (¥2700 [US $391.45] and ¥10 548 [US $1529.25], respectively). Primary cooking fuel was included as an indicator of household living environment and energy infrastructure, with clean fuels (gas, electricity, solar energy, or methane) generally more common in higher-socioeconomic households and unclean fuels (wood, straw, or coal) more common in lower-socioeconomic households.^33,34^

Child- and parent-level covariates largely reflected characteristics present at or prior to birth, whereas household-level variables were collected at the time of cognitive assessment and reflect contemporaneous household conditions. Parental education and household income were considered indicators of SES and established confounders associated with both breastfeeding practices and child cognitive development.^16,35,36^

### Statistical Analysis

Baseline characteristics were summarized using unweighted descriptive statistics by breastfeeding duration group. Temporal trends in breastfeeding duration from 1995 to 2009 were summarized using survey-weighted means across 4 birth-year intervals, overall and stratified by annual household income per capita and parental educational level. Linear trends were tested using survey-weighted linear regression, modeling breastfeeding duration (in months) against ordinal birth-year intervals. Associations between breastfeeding duration and cognitive outcomes were examined using survey-weighted generalized linear models, with Gaussian models for continuous *z* scores and logistic models for binary poor-performance outcomes. For continuous outcomes, β coefficients represent differences in test *z *scores (in SD units) comparing breastfeeding duration greater than 6 months with 6 months or less. Three nested models were fitted: unadjusted, non-SES adjusted (child sex, ethnicity, gestational age, birth order, birth year, parental age at birth, residence, and cooking fuel type), and fully SES adjusted (parental educational level and annual household income per capita). Multicollinearity among covariates was assessed using generalized variance inflation factors.

Sensitivity analyses were primarily conducted using the fully SES-adjusted model. We further adjusted the models by sequentially adding parental cognitive ability and offspring birth weight, with missing data handled using multiple imputation by chained equations. Alternative parsimonious and birth-fixed models were used to assess robustness to covariate specification and the timing of household covariate measurement. E-values were calculated to evaluate the potential bias from unmeasured confounding.^37^ Analyses using raw cognitive scores with age adjustment yielded similar results.

Nonlinear associations were explored using restricted cubic spline models with breastfeeding duration modeled continuously (≤24 months) in the fully SES-adjusted model; analyses were restricted to durations of 24 months or less to ensure estimation stability. The number of knots was selected using the Akaike information criterion. The *P* value for overall association tested whether breastfeeding duration was associated with the outcome, and the *P* value for nonlinearity tested whether the association deviated from linearity. All analyses incorporated CFPS sampling weights to account for unequal selection probabilities, nonresponse, and poststratification to national census data, thereby enabling nationally representative estimates.^38^ Statistical significance was set at *P* < .05 using 2-sided tests, with 95% CIs reported for all estimates of association. All statistical procedures were performed using R, version 4.4.2 (R Foundation for Statistical Computing).

## Results

### Baseline Characteristics

Of 6697 youths who met age eligibility and completed both tests at their first eligible wave, 78 were excluded because of missing breastfeeding information and 395 because they were never breastfed. After excluding an additional 788 individuals with missing covariates or survey design variables, the final analytic sample included 5436 youths (eFigure 1 in Supplement 1). Of these youths, 2583 (47.5%) were female and 2853 (52.5%) were male; mean (SD) age was 12.03 (1.58) years. A total of 750 youths (13.8%) were breastfed for 6 months or less and 4686 (86.2%) for more than 6 months (Table 1). Compared with youths breastfed for 6 months or less, those breastfed longer than 6 months were more often male, were more likely to belong to an ethnic minority population, and had shorter gestational age. They were also more likely to be born in earlier cohorts, reside in rural areas, and have lower household income, lower parental educational attainment, and a higher proportion of unclean cooking fuel use.

**Table 1.** Baseline Characteristics of Study Participants by Breastfeeding Duration

Characteristic	Participants, No. (%)		*P* value^a^	SMD^b^
	Breastfed ≤6 mo (n = 750)	Breastfed >6 mo (n = 4686)		
**Offspring characteristics**				
Age, mean (SD)	11.91 (1.53)	12.05 (1.59)	.03	0.09
Sex				
Female	401 (53.5)	2182 (46.6)	.001	0.14
Male	349 (46.5)	2504 (53.4)		
Ethnicity				
Han	678 (90.4)	4104 (87.6)	.03	0.09
Minority population^c^	72 (9.6)	582 (12.4)		
Gestational age				
≤9 mo	443 (59.1)	3029 (64.6)	.004	0.12
>9 mo	307 (40.9)	1657 (35.4)		
Birth order				
First	441 (58.8)	2587 (55.2)	.07	0.07
Second or later	309 (41.2)	2099 (44.8)		
Birth year				
1995-1998	207 (27.6)	1654 (35.3)	<.001	0.26
1999-2002	197 (26.3)	1427 (30.5)		
2003-2006	163 (21.7)	848 (18.1)		
2007-2009	183 (24.4)	757 (16.2)		
**Parent characteristics**				
Maternal age at birth, y				
<30	562 (74.9)	3645 (77.8)	.09	0.07
≥30	188 (25.1)	1041 (22.2)		
Paternal age at birth, y				
<30	480 (64.0)	3136 (66.9)	.13	0.06
≥30	270 (36.0)	1550 (33.1)		
Mother’s educational level, y				
<6	181 (24.1)	1464 (31.2)	<.001	0.27
6-9	419 (55.9)	2704 (57.7)		
>9	150 (20.0)	518 (11.1)		
Father’s educational level, y				
<6	99 (13.2)	810 (17.3)	<.001	0.26
6-9	454 (60.5)	3130 (66.8)		
>9	197 (26.3)	746 (15.9)		
**Family characteristics**				
Place of residence				
Rural	379 (50.5)	2976 (63.5)	<.001	0.26
Urban	371 (49.5)	1710 (36.5)		
Annual household income per capita, ¥^d^				
≤2700 (Low)	133 (17.7)	1229 (26.2)	<.001	0.29
>2700-10 548 (Middle)	353 (47.1)	2362 (50.4)		
>10 548 (High)	264 (35.2)	1095 (23.4)		
Primary cooking fuel type				
Clean	443 (59.1)	2401 (51.2)	<.001	0.16
Unclean	307 (40.9)	2285 (48.8)		

Abbreviation: SMD, standardized mean difference.

^a^ *P* values were calculated using the Student *t* test for age and the χ^2^ test for categorical variables.

^b^ SMDs were calculated to quantify the magnitude of differences in baseline characteristics, with values greater than 0.10 indicating a meaningful imbalance.

^c^ Ethnic minority groups included all non-Han ethnicities (eg, Buyi, Hui, Manchu, Miao, Yi, and others) as classified in the China Family Panel Studies dataset.

^d^ ¥2700 = US $391.45, ¥10 548 = US $1529.25.

### Temporal Trends in Breastfeeding Duration

**Figure 1.**
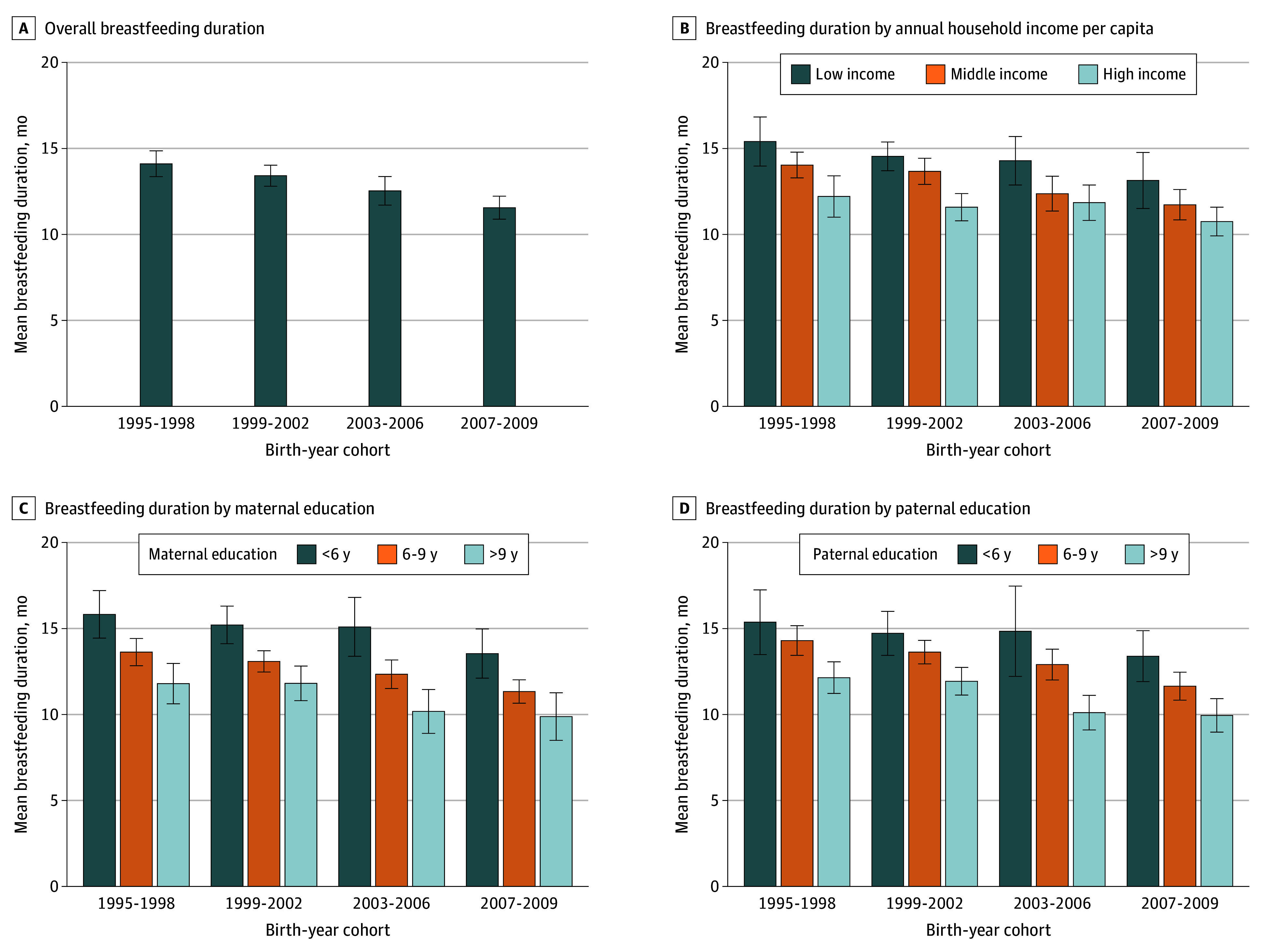
Bar Graph Showing Survey-Weighted Mean Breastfeeding Duration Overall and by Household Income and Parental Education Bars represent survey-weighted mean breastfeeding duration for each birth cohort, with error bars indicating 95% CIs. Birth cohorts were defined by birth-year intervals.

Survey-weighted analyses showed a significant decline in breastfeeding duration across successive birth cohorts from the 1995 to 1998 period (mean [SD], 14.11 [8.23] months) to the 2007 to 2009 period (mean [SD], 11.56 [6.14]) (*P* < .001 for trend) (Figure 1A and eFigure 4A in Supplement 1). When stratified by household income per capita, breastfeeding duration was consistently longer among youths from lower-income households across all birth cohorts, with significant declining trends over time in high-income (mean [SD] breastfeeding duration declined from 12.21 [6.33] to 10.75 [5.38] months; *P* = .007 for trend), middle-income (from 14.04 [8.07] to 11.73 [6.60] months; *P* < .001 for trend), and low-income (from 15.41 [9.32] to 13.14 [6.50] months; *P* = .05 for trend) groups between the 1995-1998 and 2007-2009 birth cohorts (Figure 1B and eFigure 4B in Supplement 1).

Similar patterns were observed when stratified by parental educational attainment (Figure 1C and D and eFigure 4C and D in Supplement 1). Breastfeeding duration was longer among youths with lower maternal or paternal educational level across birth cohorts, with significant declining trends over time within maternal education strata (education >9 years, mean [SD] breastfeeding duration declined from 11.80 [6.60] to 9.88 [5.00] months, *P* = .01 for trend; 6-9 years, from 13.63 [7.22] to 11.33 [5.46] months, *P* < .001 for trend; <6 years, from 15.82 [10.02] to 13.55 [8.17] months, *P* = .02 for trend) and within paternal groups with 6 years or more of education (education >9 years, from 12.14 [5.70] to 9.95 [4.82] months, *P* < .001 for trend; 6-9 years, from 14.30 [8.06] to 11.65 [6.01] months, *P* < .001 for trend), but not among those with less than 6 years of education (15.37 [10.50] to 13.39 [7.76] months, *P* = .07 for trend) between the 1995-1998 and 2007-2009 birth cohorts.

### Association Between Breastfeeding Duration and Cognitive Outcomes

**Figure 2.**
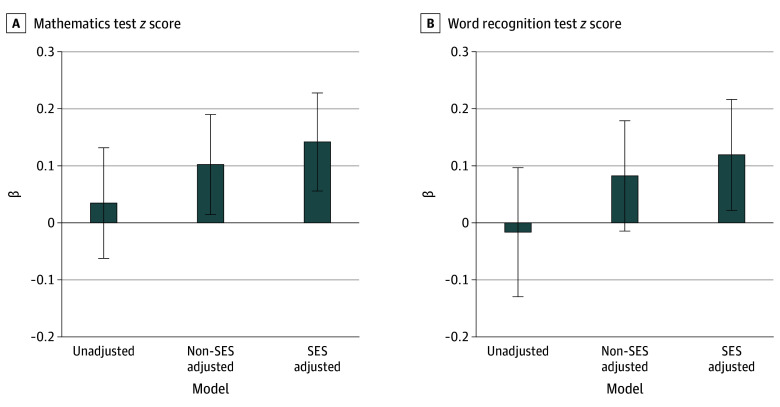
Bar Graph Showing Survey-Weighted Analysis of Breastfeeding Duration and Offspring Cognitive Performance β Indicates the difference in cognitive test *z* scores (in SD units) comparing breastfeeding duration greater than 6 months with 6 months or less. Error bars indicate 95% CIs. SES indicates socioeconomic status.

Associations between breastfeeding duration and continuous cognitive outcomes are shown in Figure 2 and eTable 1 in Supplement 1. In unadjusted analyses, breastfeeding duration of longer than 6 months was not associated with mathematics *z* scores (β = 0.03; 95% CI, −0.06 to 0.13) or word recognition *z* scores (β = −0.02; 95% CI, −0.13 to 0.09). After adjustment for non-SES covariates, breastfeeding duration of longer than 6 months was associated with higher mathematics *z* scores (β = 0.10; 95% CI, 0.01-0.19), whereas there was no association with word recognition *z* scores (β = 0.08; 95% CI, −0.02 to 0.18). In the fully SES-adjusted model, breastfeeding duration of longer than 6 months was associated with higher mathematics *z* scores (β = 0.14; 95% CI, 0.05-0.22) and higher word recognition *z* scores (β = 0.12; 95% CI, 0.02-0.21).

Results for binary cognitive outcomes are presented in Table 2. In unadjusted models, breastfeeding duration of longer than 6 months was not associated with poor mathematics performance (odds ratio [OR], 0.88; 95% CI, 0.66-1.17) or poor word recognition performance (OR, 0.88; 95% CI, 0.66-1.17). In non-SES–adjusted models, breastfeeding duration of longer than 6 months was associated with lower odds of poor mathematics (OR, 0.74; 95% CI, 0.55-0.99) and poor word recognition (OR, 0.71; 95% CI, 0.53-0.95) performance. In fully SES-adjusted models, breastfeeding duration of longer than 6 months was associated with lower odds of poor mathematics (OR, 0.65; 95% CI, 0.48-0.88) and poor word recognition (OR, 0.64; 95% CI, 0.47-0.87) performance.

**Table 2.** Survey-Weighted Association Between Breastfeeding Duration and Poor Cognitive Performance Among Offspring

Outcome, breastfeeding duration	Unadjusted model		Non-SES–adjusted model		SES-adjusted model	
	OR (95% CI)	*P* value	OR (95% CI)	*P* value	OR (95% CI)	*P* value
Poor mathematics performance						
≤6 mo	1 [Reference]	NA	1 [Reference]	NA	1 [Reference]	NA
>6 mo	0.88 (0.66-1.17)	.74	0.74 (0.55-0.99)	.04	0.65 (0.48-0.88)	.006
Poor word recognition performance						
≤6 mo	1 [Reference]	NA	1 [Reference]	NA	1 [Reference]	NA
>6 mo	0.88 (0.66-1.17)	.38	0.71 (0.53-0.95)	.02	0.64 (0.47-0.87)	.004

Abbreviations: NA, not applicable; OR, odds ratio; SES, socioeconomic status.

Results were similar after adjustment for parental cognition and offspring birth weight (eTable 3 in Supplement 1), exclusion of contemporaneous household factors (eTable 4 in Supplement 1), and use of age-adjusted raw scores (eTable 6 in Supplement 1). E-values further supported these findings (eTable 5 in Supplement 1). Additionally, variance inflation factor analyses showed no evidence of multicollinearity among covariates (all adjusted generalized variance inflation factors <2.0) (eTable 2 in Supplement 1).

### Nonlinear Association Analysis

**Figure 3.**
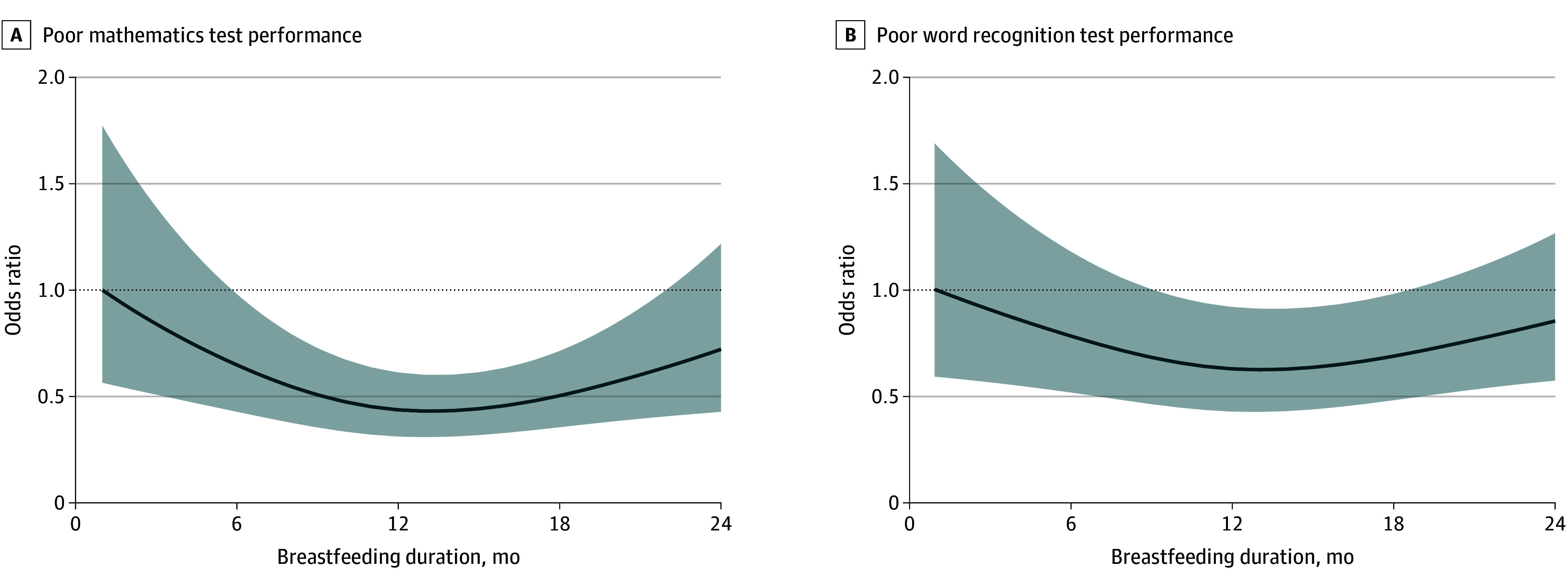
Survey-Weighted Nonlinear Analysis of Breastfeeding Duration and Poor Cognitive Performance Using Restricted Cubic Splines Shading indicates 95% CIs.

Restricted cubic spline analyses indicated nonlinear associations between some breastfeeding durations and cognitive outcomes (Figure 3). For poor mathematics performance (Figure 3A), the odds decreased with increasing breastfeeding duration up to approximately 6 to 12 months, with attenuation at longer durations. For poor word recognition performance (Figure 3B), the lowest odds were also observed around 6 to 12 months, with attenuation thereafter.

## Discussion

In this study of Chinese youths, longer breastfeeding duration was not associated with cognitive performance in the unadjusted model but was associated with better cognitive performance after adjustment for socioeconomic factors and across multiple sensitivity analyses. In addition, nonlinear analyses further suggested that the associations were not strictly linear. In this cohort, longer breastfeeding from 1995 to 2009 was more common among families with lower parental educational level and household income per capita, with most youths (86.2%) breastfed for more than 6 months. This reflects a socioeconomic distribution distinct from that observed in high-income countries.^14,21^

Survey-weighted analyses revealed a decline in breastfeeding duration across all socioeconomic groups in China from 1995 to 2009, coinciding with rapid socioeconomic transformation, including increased female labor force participation, expansion of the formula market, and intensified commercial marketing.^39,40^ Despite this overall decline, longer breastfeeding duration remained more common among families with lower income and lower parental educational attainment across birth cohorts. This distribution was likely shaped by structural factors, such as short maternity leave policies, differential access to formula, and heterogeneity in work-related constraints across socioeconomic strata.^39,41,42,43,44^ Although our analyses were limited to births between 1995 and 2009, surveillance data indicate that post-2009 breastfeeding trends in China have shown limited progress and remain below international targets, underscoring the need for sustained breastfeeding support while warranting caution in extrapolating these estimates to more recent cohorts.^9,45^

Previous studies examining associations between breastfeeding and cognitive outcomes have been challenged by confounding by socioeconomic factors across populations.^15,16,26^ In many high-income countries, higher SES is associated with both longer breastfeeding and more cognitively enriching home environments, leading to positive confounding.^26,46^ Consistent with this, the UK Millennium Cohort Study reported substantial attenuation of breastfeeding-cognition estimates after adjustment for socioeconomic and parental cognitive factors.^15^

In low- and middle-income countries, the direction of the association between SES and breastfeeding differs. In some countries (eg, Brazil), breastfeeding is distributed evenly across SES levels, with positive associations persisting after adjustment.^23^ In other countries, families with lower SES tend to breastfeed longer, yet associations with later cognitive or educational outcomes are inconsistent. A systematic review focusing on sub-Saharan Africa showed that longer breastfeeding duration was not associated with educational achievement.^21^ The Promotion of Breastfeeding Intervention Trial (PROBIT), with its rigorous experimental design, reported limited overall benefits for global cognitive outcomes at 16-year follow-up, with effects largely confined to verbal domains.^47^ Differences between PROBIT and the present findings may reflect variation in study design, outcome measures, and socioeconomic context across populations.

In our study, longer breastfeeding in China from 1995 to 2009 was more common among families with lower SES, who also had fewer resources related to early-life cognitive enrichment. This distribution would be expected to attenuate the observed estimates. Consequently, estimates for the association with cognitive performance were higher after socioeconomic adjustment, contrasting with evidence from Western populations where adjustment typically attenuated estimates.^15^

Biologic plausibility has been proposed, but causal biologic mechanisms cannot be inferred from this observational study. Emerging evidence suggests that breastfeeding may be involved in neurodevelopment and cognitive maturation through pathways involving infant gut microbiome composition and metabolic by-products via the microbiota-gut-brain axis^48,49,50^ as well as through unique bioactive compounds in breast milk that may directly support brain development and neuronal connectivity.^51,52^

Exploratory analyses using restricted cubic spline suggested nonlinear associations between breastfeeding duration and cognitive outcomes around 6 to 12 months. There was no association at longer durations, which may relate to developmental timing, changes in milk composition, or potential issues with complementary feeding (eg, delayed introduction or suboptimal quality). These factors could not be examined because detailed dietary data were unavailable. Nevertheless, longer breastfeeding was consistently associated with lower odds of poor cognitive performance compared with shorter durations, which is broadly consistent with the WHO-recommended 6-month benchmark.^4^ These findings warrant confirmation in future studies with more detailed early-life nutritional data.

Longer breastfeeding was associated with better youth cognitive performance after adjustment for SES. While modest individually, these differences (0.1-0.2 SD) represent potentially meaningful population-level shifts with implications for human capital development. As breastfeeding practices in China remain suboptimal, continued policy support is warranted. Practical measures include extended maternity leave, enhanced workplace lactation support, and stronger regulation of breast milk substitute marketing.

### Strengths and Limitations

This study has several strengths. The use of a large, nationally representative CFPS sample enhanced statistical power and generalizability. In addition, multiple sensitivity analyses were conducted to evaluate the robustness of the findings. Importantly, breastfeeding in China from 1995 to 2009 was more common among families with lower SES. This distribution differs from that in many high-income countries and provided an opportunity to examine associations under conditions of negative socioeconomic confounding.

Several limitations must be acknowledged. As an observational study, causal inference could not be established. Residual confounding and confounding by indication may remain due to missing data (eg, parental cognition), unmeasured early-life home cognitive stimulation, or misalignment between the timing of socioeconomic measurement and childbirth. However, sensitivity analyses using birth-fixed covariates and E-values suggested that results were unlikely driven by contemporary factors or moderate unmeasured confounding. In addition, retrospective breastfeeding information may introduce recall error and clustering at round numbers, although prior studies suggest reasonable accuracy.^53^ Precision was limited for gestational age because it was recorded in months rather than weeks; however, results were robust to adjustment for this variable. Finally, the inability to distinguish between exclusive and partial breastfeeding may have attenuated estimates and limited dose-response assessment.

## Conclusions

In this cross-sectional study of Chinese youths, longer breastfeeding duration was not associated with cognitive performance in the unadjusted model. However, an association with better cognitive performance was found after adjustment for socioeconomic factors in a population in which longer breastfeeding was more common among families with lower SES. Further longitudinal and mechanistic studies are needed to better understand the pathways underlying these observations.

## Notes

Supplement 1. **eFigure 1. **Flow Chart of Study Participant Selection **eFigure 2.** Distribution of Recalled Breastfeeding Duration (Months) **eFigure 3. **Directed acyclic graph illustrating covariate selection for the association between breastfeeding duration and adolescent cognitive performance **eFigure 4. **Survey-Weighted Temporal Trends in Breastfeeding Duration Across Birth Cohorts, Overall and by Socioeconomic Characteristics **eTable 1. **Survey-Weighted Association Between Breastfeeding Duration and Offspring Cognitive Performance (Continuous Outcomes) **eTable 2. **Multicollinearity Diagnostics for Covariates Included in the Fully Adjusted Survey-Weighted Regression Models **eTable 3. **Survey-Weighted Sensitivity Analyses for Associations Between Breastfeeding Duration and Offspring Cognitive Performance (Binary and Continuous Outcomes) **eTable 4. **Survey-Weighted Sensitivity Analyses for the Association Between Breastfeeding Duration (>6 Months) and Offspring Cognitive Performance Using Alternative Covariate Specifications **eTable 5. **Survey-Weighted Sensitivity Analyses Using E-values for the Association Between Breastfeeding Duration and Adolescent Cognitive Performance **eTable 6. **Survey-Weighted Association Between Breastfeeding Duration and Offspring Cognitive Test Scores (Continuous Outcomes)

Supplement 2.
Data Sharing Statement
