# Supplementary material for: Breastfeeding Duration and Cognitive Performance Among Youths
Source: JAMA Netw Open. 2026 Apr 23;9(4):e268725. doi: 10.1001/jamanetworkopen.2026.8725 (PMC13107232; doi:10.1001/jamanetworkopen.2026.8725)
Supplement: Supplement 1. — eFigure 1. Flow Chart of Study Participant Selection eFigure 2. Distribution of Recalled Breastfeeding Duration (Months) eFigure 3. Directed acyclic graph illustrating covariate selection for the association between breastfeeding duration and adolescent cognitive performance eFigure 4. Survey-Weighted Temporal Trends in Breastfeeding Duration Across Birth Cohorts, Overall and by Socioeconomic Characteristics eTable 1. Survey-Weighted Association Between Breastfeeding Duration and Offspring Cognitive Performance (Continuous Outcomes) eTable 2. Multicollinearity Diagnostics for Covariates Included in the Fully Adjusted Survey-Weighted Regression Models eTable 3. Survey-Weighted Sensitivity Analyses for Associations Between Breastfeeding Duration and Offspring Cognitive Performance (Binary and Continuous Outcomes) eTable 4. Survey-Weighted Sensitivity Analyses for the Association Between Breastfeeding Duration (>6 Months) and Offspring Cognitive Performance Using Alternative Covariate Specifications eTable 5. Survey-Weighted Sensitivity Analyses Using E-values for the Association Between Breastfeeding Duration and Adolescent Cognitive Performance eTable 6. Survey-Weighted Association Between Breastfeeding Duration and Offspring Cognitive Test Scores (Continuous Outcomes) [file jamanetwopen-e268725-s001.pdf]

Supplemental Online Content

Tang X, Qiu Y, Qin Z, et al. Breastfeeding duration and cognitive performance among youths. *JAMA Netw Open*. 2026;9(4):e268725. doi:10.1001/jamanetworkopen.2026.8725

eFigure 1. Flow Chart of Study Participant Selection
eFigure 2. Distribution of Recalled Breastfeeding Duration (Months)
eFigure 3. Directed acyclic graph illustrating covariate selection for the association between breastfeeding duration and adolescent cognitive performance
eFigure 4. Survey-Weighted Temporal Trends in Breastfeeding Duration Across Birth Cohorts, Overall and by Socioeconomic Characteristics
eTable 1. Survey-Weighted Association Between Breastfeeding Duration and Offspring Cognitive Performance (Continuous Outcomes)
eTable 2. Multicollinearity Diagnostics for Covariates Included in the Fully Adjusted Survey-Weighted Regression Models
eTable 3. Survey-Weighted Sensitivity Analyses for Associations Between Breastfeeding Duration and Offspring Cognitive Performance (Binary and Continuous Outcomes)
eTable 4. Survey-Weighted Sensitivity Analyses for the Association Between Breastfeeding Duration (>6 Months) and Offspring Cognitive Performance Using Alternative Covariate Specifications
eTable 5. Survey-Weighted Sensitivity Analyses Using E-values for the Association Between Breastfeeding Duration and Adolescent Cognitive Performance
eTable 6. Survey-Weighted Association Between Breastfeeding Duration and Offspring Cognitive Test Scores (Continuous Outcomes)

This supplemental material has been provided by the authors to give readers additional information about their work.

© 2026 Tang et al. *JAMA Network Open*.

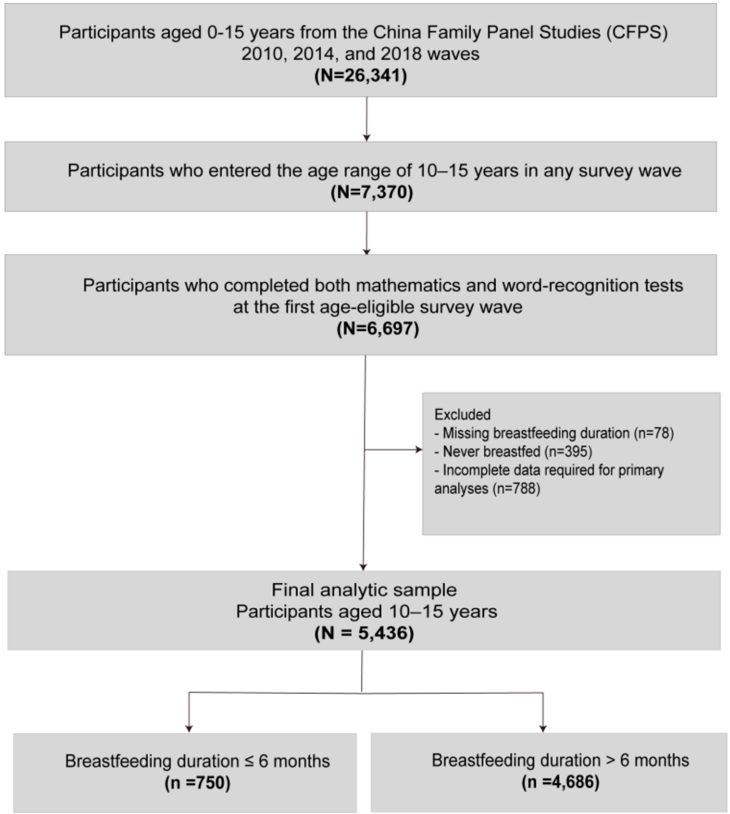

eFigure 1. Flow Chart of Study Participant Selection.

Notes: The flowchart illustrates the selection of the analytic sample from the China Family Panel Studies (CFPS) 2010, 2014, and 2018 waves. Adolescents were eligible if they entered the age range of 10–15 years in any survey wave. For each participant, the first survey wave in which age eligibility was met was defined as the baseline for cognitive assessment. Participants who completed both mathematics and word-recognition tests at this first age-eligible wave were retained. Individuals with missing breastfeeding duration information, those who were never breastfed, and those with incomplete data required for the primary analyses were excluded. The final analytic sample comprised 5,436 adolescents.

© 2026 Tang et al. *JAMA Network Open*.

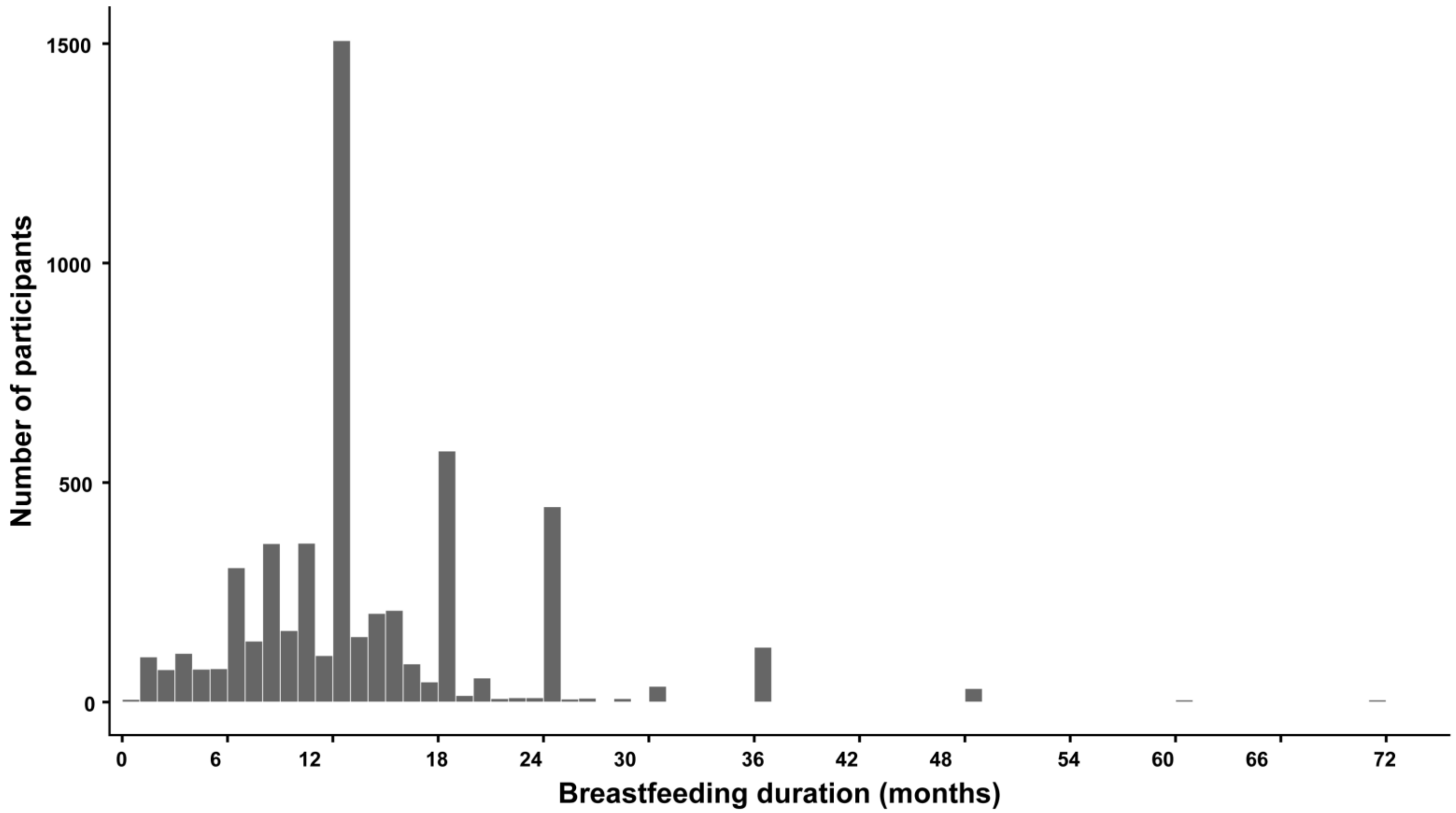

eFigure 2. Distribution of Recalled Breastfeeding Duration (Months).
Notes: The histogram displays the distribution of breastfeeding duration (in months) as recalled by caregivers. Peaks observed at 6, 12, 18, and 24 months indicate clustering (digit preference) typical of retrospective reporting. This distribution characteristic was considered when interpreting non-linear associations.

© 2026 Tang et al. *JAMA Network Open*.

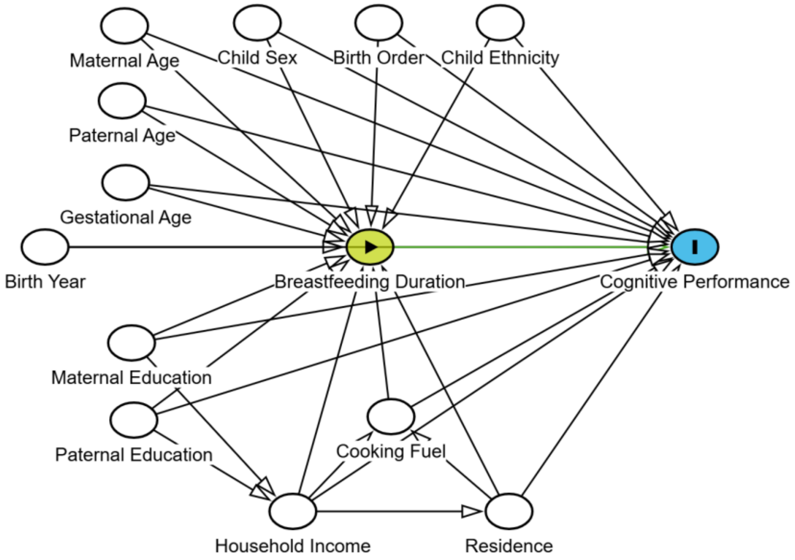

eFigure 3. Directed acyclic graph illustrating covariate selection for the association between breastfeeding duration and adolescent cognitive performance.

Notes: This DAG depicts hypothesized causal relationships among breastfeeding duration (green node, exposure), adolescent cognitive performance (blue node, outcome), and covariates (white nodes). Variables are arranged by domain: biological and demographic factors (top), birth cohort trends (left), and socioeconomic pathways (bottom). Arrows represent assumed causal directions based on prior epidemiologic literature and biological plausibility. Algorithmic analysis using DAGitty confirmed that the covariates included in the fully adjusted models constitute a minimal sufficient adjustment set for estimating the total association with minimal confounding.

© 2026 Tang et al. *JAMA Network Open*.

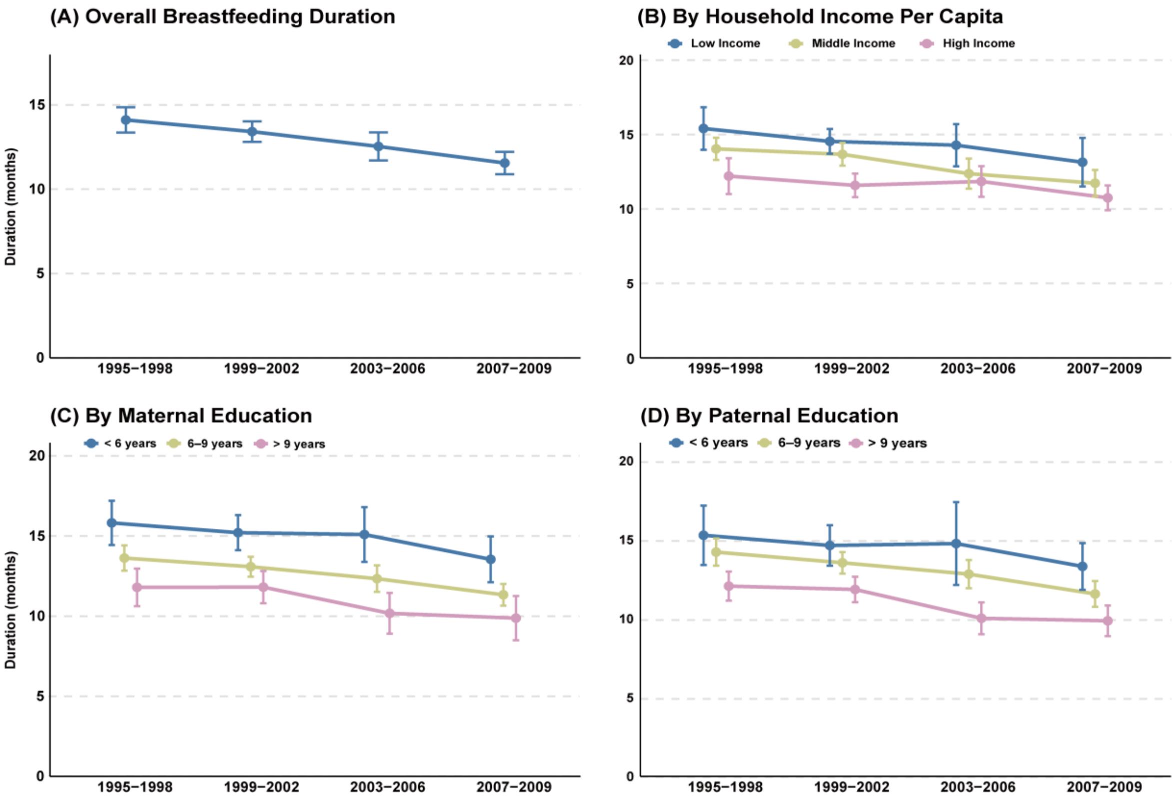

eFigure 4. Survey-Weighted Temporal Trends in Breastfeeding Duration Across Birth Cohorts, Overall and by Socioeconomic Characteristics.
Notes: Lines represent survey-weighted mean breastfeeding duration (months) across birth cohorts, with shaded areas indicating 95% confidence intervals. Birth cohorts were defined by birth-year intervals (1995–1998, 1999–2002, 2003–2006, and 2007–2009). Panel A shows trends for the overall sample; Panels B–D show trends stratified by household income per capita, maternal education, and paternal education, respectively. Linear trends across birth cohorts were evaluated using survey-weighted linear regression models with birth cohort specified as an ordinal predictor.

© 2026 Tang et al. *JAMA Network Open*.

eTable 1. Survey-Weighted Association Between Breastfeeding Duration and Offspring Cognitive Performance (Continuous Outcomes).

	Unadjusted Model		Non-SES Adjusted Model		SES-Adjusted Model	
	β (95%CI)	P value	β (95%CI)	P value	β (95%CI)	P value
Mathematics test z score						
⩽6 months	1.00 (Ref)		1.00 (Ref)		1.00 (Ref)	
>6 months	0.03 (-0.06, 0.13)	.51	0.10 (0.01, 0.19)	.03	0.14 (0.05, 0.22)	.002
Word-recognition test z score						
⩽6 months	1.00 (Ref)		1.00 (Ref)		1.00 (Ref)	
>6 months	-0.02 (-0.13, 0.09)	.75	0.08 (-0.02, 0.18)	.10	0.12 (0.02, 0.21)	.02

Notes: CI, confidence interval; SES, socioeconomic status; Ref, reference group. Data are presented as β coefficients with 95% CI from survey-weighted linear regression models for continuous outcomes (z scores).

© 2026 Tang et al. *JAMA Network Open*.

eTable 2. Multicollinearity Diagnostics for Covariates Included in the Fully Adjusted Survey-Weighted Regression Models.

	Word-recognition test z score	Mathematics test z score
Covariate	GVIF^(1/(2×Df))	GVIF^(1/(2×Df))
Breastfeeding duration (>6 vs ≤6 months)	1.25	1.23
Birth cohort	1.19	1.14
Sex	1.09	1.15
Birth order	1.43	1.32
Gestational age	1.25	1.11
Ethnicity	1.31	1.33
Father's age at birth	1.66	1.54
Mother's age at birth	1.41	1.61
Maternal education	1.27	1.24
Paternal education	1.26	1.14
Household income group	1.22	1.18
Urban residence	1.31	1.18
Cooking fuel type	1.14	1.24

Notes: All generalized variance inflation factors (GVIF^(1/(2×Df))) were <2, indicating no evidence of severe multicollinearity.

© 2026 Tang et al. *JAMA Network Open*.

eTable 3. Survey-Weighted Sensitivity Analyses for Associations Between Breastfeeding Duration and Offspring Cognitive Performance (Binary and Continuous Outcomes).

	Adjusting for maternal cognitive ability		Adjusting for paternal cognitive ability		Adjusting for birth weight	
	Estimate (95%CI)	P value	Estimate (95%CI)	P value	Estimate (95%CI)	P value
Poor mathematics performance [a]						
≤6 months	1.00 (Ref)		1.00 (Ref)		1.00 (Ref)	
>6 months	0.64 (0.47, 0.87)	.005	0.66 (0.48, 0.89)	.007	0.65 (0.48, 0.89)	.006
Poor word-recognition performance [a]						
≤6 months	1.00 (Ref)		1.00 (Ref)		1.00 (Ref)	
>6 months	0.64 (0.47, 0.86)	.004	0.65 (0.48, 0.89)	.007	0.65 (0.48, 0.88)	.005
Mathematics test z score [b]						
≤6 months	1.00 (Ref)		1.00 (Ref)		1.00 (Ref)	
>6 months	0.15 (0.06, 0.23)	<.001	0.13 (0.05, 0.21)	.002	0.14 (0.05, 0.22)	.002
Word-recognition test z score [b]						
≤6 months	1.00 (Ref)		1.00 (Ref)		1.00 (Ref)	
>6 months	0.12 (0.02, 0.21)	.01	0.10 (0.01, 0.20)	.03	0.11 (0.02, 0.21)	.02

Notes: CI, confidence interval; Ref, reference group. All sensitivity analyses were conducted based on the fully SES-adjusted model. Model 1 was additionally adjusted for maternal cognitive ability; Model 2 for paternal cognitive ability; and Model 3 for birth weight. The proportions of missing data were 16.80% for maternal cognitive ability (913/5436), 26.32% for paternal cognitive ability (1431/5436), and 15.62% for birth weight (849/5436). Binary outcomes ([a]) were analyzed using survey-weighted logistic regression models; continuous outcomes ([b]) were analyzed using survey-weighted linear regression models.

© 2026 Tang et al. *JAMA Network Open*.

eTable 4. Survey-Weighted Sensitivity Analyses for the Association Between Breastfeeding Duration (>6 Months) and Offspring Cognitive Performance Using Alternative Covariate Specifications.

	Parsimonious (core) model		Birth-fixed model	
	Estimate (95%CI)	P value	Estimate (95%CI)	P value
Poor mathematics performance [a]				
≤6 months	1.00 (Ref)		1.00 (Ref)	
>6 months	0.66 (0.48, 0.89)	.008	0.69 (0.52, 0.92)	.01
Poor word-recognition performance [a]				
≤6 months	1.00 (Ref)		1.00 (Ref)	
>6 months	0.64 (0.47, 0.87)	.005	0.67 (0.49, 0.91)	.01
Mathematics test z score [b]				
≤6 months	1.00 (Ref)		1.00 (Ref)	
>6 months	0.14 (0.05, 0.23)	.002	0.12 (0.04, 0.21)	.005
Word-recognition test z score [b]				
≤6 months	1.00 (Ref)		1.00 (Ref)	
>6 months	0.12 (0.02, 0.22)	.02	0.10 (0.01, 0.20)	.04

Notes: CI, confidence interval; Ref, reference group. Parsimonious models were adjusted for birth year, child sex, birth order, gestational age, ethnicity, maternal educational attainment, household income per capita, and place of residence. Birth-fixed models were adjusted only for covariates plausibly determined at or prior to birth, including birth year, child sex, ethnicity, birth order, gestational age, parental age at birth, and parental educational attainment. Binary outcomes ([a]) were analyzed using survey-weighted logistic regression models; continuous outcomes ([b]) were analyzed using survey-weighted linear regression models.

© 2026 Tang et al. *JAMA Network Open*.

eTable 5. Survey-Weighted Sensitivity Analyses Using E-values for the Association Between Breastfeeding Duration and Adolescent Cognitive Performance.

	Estimate (95%CI)	P Value	E-Value (Point)	E-Value (Lower CI)
Poor mathematics performance [a]				
≤6 months	1.00 (Ref)			
>6 months	0.65 (0.48, 0.88)	.006	2.43	1.52
Poor word-recognition performance [a]				
≤6 months	1.00 (Ref)			
>6 months	0.64 (0.47, 0.87)	.004	2.49	1.57
Mathematics test z score [b]				
≤6 months	1.00 (Ref)			
>6 months	0.14 (0.05, 0.22)	.002	1.53	1.28
Word-recognition test z score [b]				
≤6 months	1.00 (Ref)			
>6 months	0.12 (0.02, 0.21)	.02	1.46	1.16

Notes: CI, confidence interval. E-value (point) represents the minimum strength of association that an unmeasured confounder would need to have with both the exposure and the outcome to fully explain the observed association. E-value (lower CI) represents the minimum strength of association required for an unmeasured confounder to shift the lower bound of the confidence interval to include the null value. Binary outcomes ([a]) were analyzed using survey-weighted logistic regression models; continuous outcomes ([b]) were analyzed using survey-weighted linear regression models.

© 2026 Tang et al. *JAMA Network Open*.

eTable 6. Survey-Weighted Association Between Breastfeeding Duration and Offspring Cognitive Test Scores (Continuous Outcomes).

	Unadjusted Model		Non-SES Adjusted Model		SES-Adjusted Model	
	β (95%CI)	P value	β (95%CI)	P value	β (95%CI)	P value
Mathematics test score (raw)						
⩽6 months	1.00 (Ref)		1.00 (Ref)		1.00 (Ref)	
>6 months	0.36 (−0.06, 0.78)	.09	0.37 (0.09, 0.66)	.011	0.51 (0.23, 0.79)	<.001
Word-recognition test score (raw)						
⩽6 months	1.00 (Ref)		1.00 (Ref)		1.00 (Ref)	
>6 months	0.09 (−0.67, 0.85)	.81	0.50 (−0.11, 1.12)	.11	0.74 (0.12, 1.36)	.02

Notes: CI, confidence interval; SES, socioeconomic status; Ref, reference group. Data are presented as β coefficients with 95% confidence intervals from survey-weighted linear regression models for continuous outcomes (unstandardized mathematics and word-recognition test scores). The non–SES adjusted and SES-adjusted models were additionally adjusted for offspring age; the unadjusted model included breastfeeding duration only.

© 2026 Tang et al. *JAMA Network Open*.
