# Supplementary material for: Breastfeeding Duration and Cognitive Performance Among Youths
Source: JAMA Netw Open. 2026 Apr 23;9(4):e268725. doi: 10.1001/jamanetworkopen.2026.8725 (PMC13107232; doi:10.1001/jamanetworkopen.2026.8725)
Supplement: Supplement 2. — Data Sharing Statement [file jamanetwopen-e268725-s002.pdf]

Data Sharing Statement

Tang. Breastfeeding Duration and Cognitive Performance Among Youths. *JAMA Netw Open*. Published April 23, 2026. doi:10.1001/jamanetworkopen.2026.8725

Data
Data available: No
